# L-5-[^11^C]-glutamine PET of breast cancer: Preclinical studies in mouse models

**DOI:** 10.1016/j.nucmedbio.2025.109092

**Published:** 2025-08-30

**Authors:** Christopher T. Hensley, Prashanth Padakanti, Raheema Damani, Christina Dulal, Hoon Choi, Shihong Li, Jianbo Cao, Hsiaoju Lee, Austin Pantel, Elizabeth Li, David Mankoff, Rong Zhou

**Affiliations:** a Department of Radiology, University of Pennsylvania, Philadelphia, PA, USA; b Cancer Research UK, University of Cambridge, Cambridge, UK; c Department of Radiology, University of Alabama at Birmingham, Birmingham, AL, USA

**Keywords:** *L*-5-[^11^C]-glutamine, *L*-5-[^11^C]-glutamate, Glutaminase, Cancer, Metabolism

## Abstract

**Background:**

Glutamine is an important metabolic substrate in many aggressive tumors, with comparable importance to glucose metabolism. Utilizing human breast cancer mouse xenograft models, we studied the kinetics of the PET imaging agent, L-5-[^11^C]-glutamine ([^11^C]glutamine or [^11^C]GLN) a biochemical authentic substrate for glutamine metabolism, to further characterize the metabolism of glutamine and downstream labeled metabolites. Studies were performed with and without inhibition of the enzyme, glutaminase (GLS), the first step in glutamine catabolism that generates glutamate, and key target for therapy directed to glutamine-metabolizing cancers.

**Methods:**

The study used xenograft mouse models for two breast cancer cell lines, HCC1806, a highly glutaminolytic triple-negative cell line, and MCF-7, a hormone receptor positive line with only low levels of glutaminolysis. Mice were injected with [^11^C]glutamine and either underwent metabolite analysis or dynamic PET imaging. The contributions of individual metabolites to the total ^11^C-activity signal in blood and tumor tissue were measured at 10, 20, and 30 min *via* HPLC. We measured fractional activity in the form of [^11^C]glutamine *versus* labeled metabolites, focusing on L-5-[^11^C]-glutamate ([^11^C]glutamate or [^11^C]GLU), and any activity in the other metabolite small molecules labeled with ^11^C (^11^C-other or ^11^C-OTH). Additionally, the contribution of [^11^C] CO_2_ to total ^11^C-activity was measured. Together with image-based uptake curves, this generated estimated time activity curves for [^11^C]glutamine and downstream metabolites in both xenograft models treated with vehicle or GLS inhibitor (CB-839).

**Results:**

We found that, out to 30 min post-injection, the majority of radioactivity in highly glutaminolytic tumors (HCC1806) was in the form of [^11^C]glutamine and [^11^C]glutamate, with relatively low amounts of radioactivity in metabolites downstream of glutamate including [^11^C]CO_2_. In HCC1806 tumors, [^11^C]glutamate was retained in the large cellular glutamate pool leading to a majority fraction of total radioactivity in tumor tissue that is greater than the fraction within the blood, with this tumoral fractional pattern reversing with CB-839. This phenomenon leads to a total tumor time-activity curve that is only marginally different before and after CB-839. The radioactivity patterns of MCF-7 tumors after vehicle treatment were similar HCC1806 tumors after CB-839 treatment.

**Conclusion:**

Our studies on [^11^C]glutamine in breast cancer models show significant retention of ^11^C-activity in the form of [^11^C]glutamate in tumors with high GLS activity that confounds non-invasive inference of GLS activity. This suggests limited utility for [^11^C]glutamine PET for inferring tumor GLS activity and its specific antagonism by drug inhibitors. Our analysis of labeled metabolites in mouse models does, however, yield insights that include the retention of glutamate generated by GLS-mediated catabolism in a large cellular pool and also provide data that is the basis for a compartmental model of glutamine metabolism that is the subject of a companion paper.

## 1. Introduction

Glutamine (GLN) is the most abundant amino acid present in human plasma. A subset of cancers, including triple negative breast cancer (TNBC), demonstrate glutamine “addiction” where glutamine is presumed to serve as an alternative to glucose as a source of energy and building blocks for biosynthesis [1–6]. Intracellular GLN can be converted to glutamate (GLU) in the presence of the glutaminase enzyme (GLS), a rate-limiting step for glutamine catabolism, which is encoded by either the kidney-type gene (*GLS1*) or the liver-type gene (*GLS2*) [7]. Expression levels of the mitochondrial splice variant isoform of the kidney-type glutaminase (*GAC*) as well as total glutaminase activity have been demonstrated to be highly correlated to glutamine dependence in breast cancer cell lines *in vitro* [8,9]. In breast cancer, this dependence on GLS is most prevalent in TNBC, when compared to hormone receptor positive (HR+) cell lines (estrogen or progesterone receptor positive, ER+ or PR+) [8].

CB-839 (telaglenastat) is a selective inhibitor of GLS1 that has demonstrated safety and favorable PK/PD profiles in phase I studies in a variety of treatment-refractory solid tumors, including TNBC, but has failed to show the anticipated efficacy in tumor response, suggesting a need for better selective markers [10]. Responding to this need, glutamine positron emission tomography (PET) tracers have been developed to image glutamine metabolism *in vivo*, including glutamine analog 4-(2*S*,4*R*)-[^18^F]fluoroglutamine ([^18^F]fluoroglutamine or [^18^F]F-GLN) and radio-labeled authentic glutamine, L-5-[^11^C]-glutamine ([^11^C] glutamine or [^11^C]GLN) [11]. We have previously assessed the utility of [^18^F]fluoroglutamine in human breast cancer xenograft models to detect changes of GLS activity in response to CB-839 treatment. In this setting, xenografts from a TNBC line with high intrinsic GLS activity (HCC1806) were compared to those from an ER+ line (MCF-7) with much lower GLS activity. An increase in the tumor to blood ratio of [^18^F]fluoroglutamine PET, reflecting an increase in cellular glutamine concentrations with the inhibition of glutamine catabolism by GLS, was found to be a robust marker of the pharmacodynamic impact of CB-839 mediated GLS inhibition. These findings were consistent with studies showing minimal metabolism of [^18^F]fluoroglutamine, supporting its ability to assess cellular pool size provided by estimates of [^18^F]fluoroglutamine distribution volume and a reliable estimate of glutamine pool size changes as surrogate of *GLS* inhibition [12,13].

Carbon-11 labeled glutamine ([^11^C]glutamine) provides a PET radiopharmaceutical with metabolism identical to native glutamine and has been tested in pre-clinical models and early human studies [14,15]. PET-derived measures of [^11^C]glutamine may therefore provide an attractive means of estimating glutamine metabolic flux through GLS *via* tracer kinetic analysis in analogy to methods used for ^11^C-labeled glucose and thymidine tracers [16,17]. However, like other metabolic substrates such as glucose and thymidine, the rapid metabolism of glutamine into a variety of labeled downstream metabolites poses a challenge to interpreting PET images, where total ^11^C-activity cannot determine the proportional contributions of parent tracer *versus* downstream labeled metabolites. We therefore undertook a pre-clinical study of dynamic [^11^C]glutamine PET imaging along with radiometabolite analysis of blood and tumor sampled at various time points after tracer injection and tumor GLS activity using the same breast cancer mouse models previously used to characterize [^18^F]fluoroglutamine kinetics [12,13]. We hypothesized that radiometabolite analysis would guide the interpretation of dynamic [^11^C]glutamine PET signal. Additionally, we hypothesized that the ability to compare [^11^C]glutamine to prior [^18^F] fluoroglutamine results would provide a unique mechanistic insight into the underlying biology of tumor glutamine metabolism and guide the interpretation of glutamine PET imaging studies as assays of *in vivo* GLS activity.

## 2. Materials and methods

### 2.1. Materials

The standard compounds (L-glutamine, L-glutamic acid, HPLC grade methanol, acetonitrile, isopropyl alcohol, sodium bicarbonate, sodium hydroxide, hydrochloric acid) and Supelco brand Astec CHIROBIOTIC T 5um HPLC column 250 * 4.6 mm. were purchased from Sigma-Aldrich corporation, USA. DIUF water, was purchased from Fisher scientific, USA. L-5-[^13^C]-glutamine ([^13^C]glutamine or [^13^C]GLN) was purchased from Cambridge Isotope Laboratories. All the chemicals were used without any further purification. Sparge needles (18G × 1–1/2) were purchased from W.W. Grainger, Inc. USA. A 1200 series HPLC (from Agilent Technologies), Precellys Evolution homogenizer (from Bertin Technologies, Rockville, MD), 2480 series gamma counter (from Perkin Elmer, Waltham, MA) were used for these experiments.

### 2.2. Mouse models of human breast cancer

[^11^C]glutamine imaging and metabolite studies were performed in two different breast cancer xenograft mouse models used in prior studies of 4-(2*S*,4*R*)-[^18^F]fluoroglutamine PET imaging at our center [12]. This model composes of HCC1806, a highly glutaminolytic TNBC cell line, and MCF-7, an estrogen receptor + (ER+) cell line with low glutaminase activity, in the presence or absence of CB-839 (kindly provided by Calithera Biosciences, South San Francisco, CA). All animal experiments were conducted under protocols approved by University of Pennsylvania Institutional Animal Care and Use Committee. HCC1806 and MCF-7 xenograft mice were treated with CB-839 at 200 mg/kg or vehicle (VEH) by oral gavage twice daily for 2d. Either before or after the treatment, mice were injected with [^11^C]glutamine and imaged as described below. For comparisons of pre-therapy to post-therapy, the same mouse was imaged at 0 h for the pre-therapy imaging session and re-imaged at 48 h for the post-therapy imaging session.

### 2.3. Radiopharmaceutical synthesis

L-5-[^11^C]-glutamine was synthesized using previously published methodologies [14,18]. Passing criteria for radiochemical purity was >90 %. Molar activity (A_m_) was estimated at 259 GBq/μmol (7000 mCi/μmol) at the end of bombardment. Decay corrected molar activity at the end of synthesis was >565 mCi/μmol.

### 2.4. In vivo PET imaging studies and imaging analysis

**Fig. 1.**
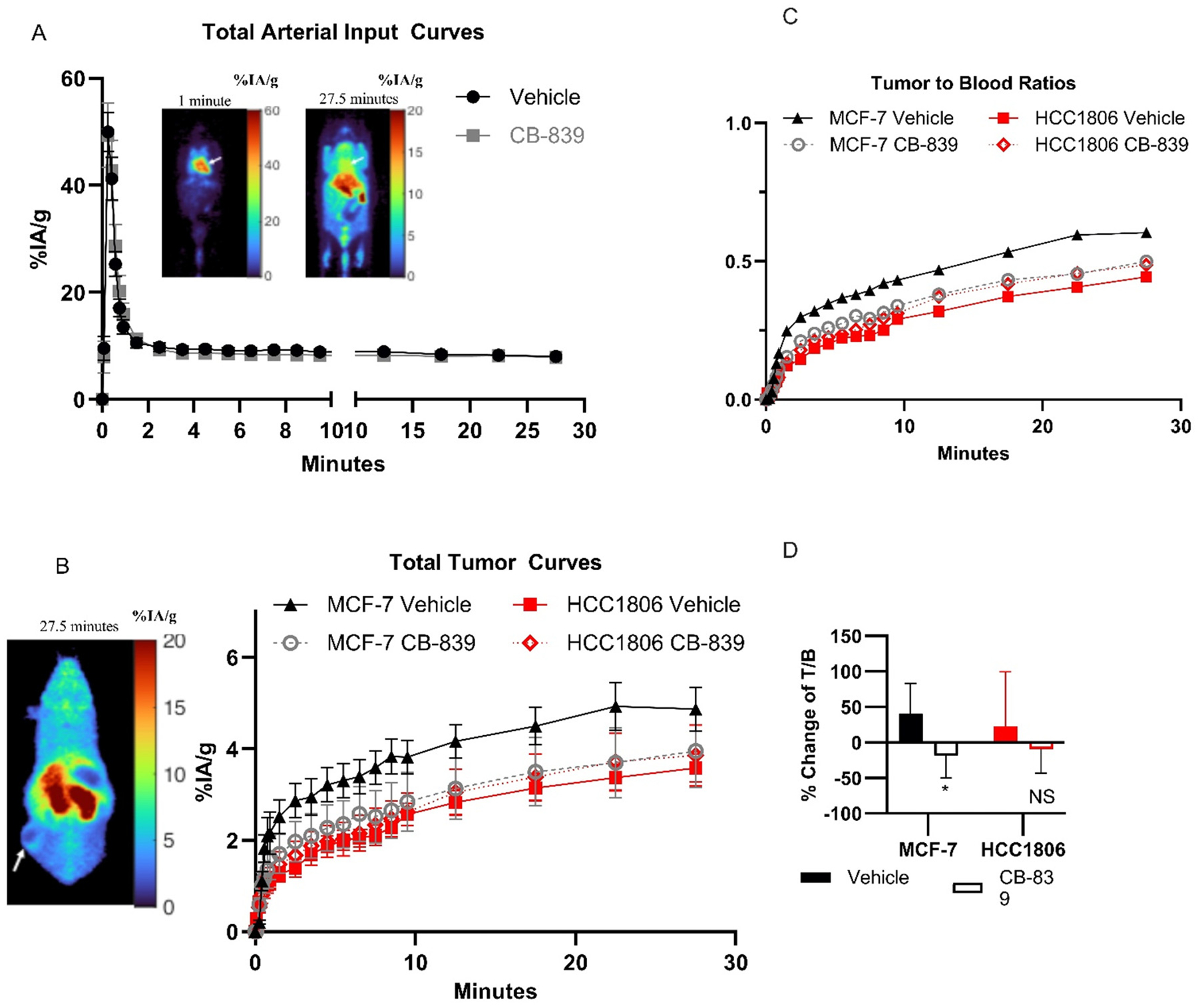
PET/CT imaging of breast cancer cell line flank xenograft-bearing mice administered a bolus of L-[5-^11^C]glutamine via tail vein injection. (A–C) Dynamic imaging was performed with the following temporal resolution- 10 s × 6 frames, 1 min × 9 frames, 5 min × 4 frames. Slice showing the blood pool in early frame and at late frame are displayed in (A) and slice showing the tumor at late timepoint is shown in (B) for reference mouse. Dynamic windowing is used to show image scale. A) Total arterial input time activity curves, comparing vehicle (n = 16) to CB-839 treated (n = 12) mice. B) Total tumor time activity curves, comparing vehicle to CB-839 treated mice, separated by MCF-7 (veh n = 9, CB-839 n = 7) and HCC1806 (veh n = 9, CB-839 n = 7) cohorts. C) Tumor to blood ratios of figure 1B to A. D) % Change of tumor to blood ratios of %IA/g, comparing static images of final timepoints of post-treatment cohort to pre-treatment cohorts (in pre-treatment cohorts, n = 7 in all conditions except MCF-7 CB-839 n = 6). In A) and B), only post-treatment cohorts are compared. NS = not significant. *p < 0.05 utilizing a paired, 2-sample t-test. Values are plotted as averages with error bars representing ±standard error of the mean.

Dynamic PET scans were conducted on a dedicated small animal scanner (A-PET), as previously described [12,19,20]. Each scan was started immediately after 1.184–16.502 MBq (0.032–0.446 mCi) injection into the mice *via* tail vein injection under 2–3 % Isofluorane/oxygen anesthesia, with a temporal resolution of 10 s × 6 frames, 1 min × 9 frames, 5 min × 4 frames, for 30 min of imaging. PET images were analyzed using MIM software version 7.1.5. To quantify the total blood clearance curve, a region of interest (ROI) of fixed size (2 × 2 × 2 mm^3^ sphere) was placed on the anatomical location of the heart (left ventricle) using the early dynamic imaging time points, noting that tracer accumulation in myocardium did not exceed blood pool background by visually examining the time course of cardiac region tracer uptake, especially in images at the end of dynamic imaging period (Fig. 1A). For tumors, ROIs were manually drawn around the entire tumor and an automated workflow produced a single spherical ROI with a volume of 0.016 cm^3^ which was centered on the tumor area with maximal uptake. This approach is akin to the “peak” ROI used in clinical PET analysis and in our prior analysis of [^18^F]fluoroglutamine [13,21]. Time-Activity Curves (TAC) of the tumor and blood were constructed as tumor to blood ratios (T/B) or % Injected Activity per gram (%IA/g), calculated from using the whole mouse counts as the reference for injected activity (Eq. (1)) [12].

(1) %IAg=Activitytumor*VolumetumorActivityWholeMouse*VolumeWholeMouse*1Volumetumor*1gcm3*1000mm3cm3*100%

where the first fraction represents the tumor fractional injected activity (IA) calculated as the ratio of the decay-corrected total tumor activity at each time point divided by the whole mouse total activity at the reference time. The latter terms represent the tumor tissue mass based on the tumor ROI volume (in mm^3^) and using assumed tissue density of 1 g/cm^3^.

The total ^11^C blood clearance curve for each mouse was corrected for partial volume effects to account for spill-out from the blood pool ROI. Previously, using a Data Spectrum Micro Deluxe phantom, a contrast recovery coefficient (CRC) curve was estimated [19]. The CRC applied here was determined for a 2-mm spherical ROI which is a typical mouse left ventricular size. Since the data within treatment groups were averaged, each total blood clearance curve was also corrected for time delay in tracer arrival with the leading-edge method [22], to align blood and tissue time-activity curves across acquisitions. We compared image-derived T/B *versus* the ratio of dissected tumor tissue after euthanasia to extracted blood samples measured *via* well counter, to validate the scaling of our image-derived blood clearance and tumor curves and further confirm that the image-derived curves preserved the correct total radioactivity levels.

### 2.5. Radioactivity extraction and metabolite analysis

At specified time points after tracer injection (t=10,20 and 30 min p.i.) the mice were sacrificed to collect blood and tissue for metabolite analysis and radioactivity counting. Blood was collected *via* cardiac puncture and tumor was harvested. For blood, red blood cell (RBC) and plasma were separated by centrifugation (3000g for 5 min at 4 °C). For tumor, the tissue was first cut into small pieces and 1 mL ice-cold PBS was added in a 2 mL tube containing ceramic beads. The tumor was then homogenized at 4 °C on a Precellys Evolution homogenizer and 1 mL ice-cold acetonitrile was added to the tissue homogenates and plasma to denature the proteins and the mixture was vortexed for 10 s followed by centrifugation (3000g for 5 min at 4 °C). The supernatant and pellet were separated, and their radioactivity was measured *via* gamma counter to provide estimates of radioactivity in the form of soluble metabolites (supernatant) *versus* macromolecules such as peptides and proteins (pellet). To obtain the absolute values for the radioactive components, the gamma counter was calibrated and the radioactivity measured on the gamma counter for the various components was quantified. Radioactivity of the components was obtained as counts per minute (CPM) from the gamma counter. These values were converted to kBq and decay corrected to time of injection and then converted to %IA/g of organ (blood or tumor). It is to be noted that only part of the tumor and blood was used for these studies and different mice (tumors) were used for different time points.

### 2.6. HPLC method for [^11^C]glutamine, [^11^C]glutamate, and other ^11^C metabolite (^11^C-other) information

An aliquot (100–200μl) of supernatant extracted from plasma and tumor was injected onto HPLC. The mobile phase used was 80:20 methanol: DIUF water at a flow rate of 1 mL/min. Stationary phase was Supelco brand Astec CHIROBIOTIC T 5um HPLC column 250 * 4.6 mm. Standards of l-glutamine and L-glutamate samples were injected onto the HPLC to find the retention times (around 3 min for L-glutamate and around 6 min for l-glutamine). The amount of the radioactivity in these samples was very low and could not be detected using the built-in radioactivity detector of the HPLC used for these experiments. Therefore, after injecting the sample onto HPLC, one-minute fractions were collected in test tubes for 14 min. The 14 fractions collected for each sample injection on HPLC were measured on a gamma counter to obtain CPM values which were plotted in a graph (Supplemental Fig. 1). The relative radioactivity values were obtained (Eqs. (2)–(4)) by summing the counts from the fractions collected for [^11^C]glutamine (CPMC11GLN), [^11^C]glutamate (CPMC11GLU), and the remainder of the radioactivity peaks (referred to as ‘other’, CPMC11OTH) over total radioactivity (${CPM}_{\text{Total}}=\text{summed}$ radioactivity of all 14 fractions collected for each sample injection). This gave us the fractional radioactive species (f(p,T),C11GLN,f(p,T),C11GLU or f(p,T),C11OTH) present in both blood and tumor supernatants.


(2) f(p,T),C11GLN=CPMC11GLNCPMTotal

(3) f(p,T),C11GLU=CPMC11GLUCPMTotal

(4) f(p,T),C11OTH=CPMC11OTHCPMTotal

### 2.7. [^11^C]CO_2_ estimation: in vivo studies

In order to estimate [^11^C]CO_2_ (volatile), two test tubes (one for acid and one for base solution) for each blood and tumor sample were prepared and labeled as acid blood, base blood, acid tumor and base tumor, following procedures similar previously described methods [23]. To the acid test tubes 1.5 mL isopropyl alcohol and 0.5 mL of 0.9 M sodium bicarbonate solution was added and the pre-weight of the tubes were noted. To the base test tubes 1.5 mL isopropyl alcohol, 0.5 mL of 0.9 M sodium bicarbonate solution and 0.5 mL of 0.1 N sodium hydroxide solution was added and the pre-weight of the tubes were noted. Each sample (blood or tumor) was divided into two parts and weighed: one immersed in a tube containing acid and the other in base tube. The post-weight of both the acid and base tubes were noted, and the net weight of the blood or tumor in each tube was calculated by subtracting the pre-weight from post-weight of the tubes. To the acid tubes, 0.5 mL of 6 M hydrochloric acid was added to protonate the carbonate present in the solution and sparged for at least 10 min to remove any free carbon dioxide present in the tubes. The base tubes will retain any carbon dioxide present in the tube in the form of sodium bicarbonate. To retain all the radioactivity present, sparging was performed carefully not to spill any liquid outside the tubes. After sparging, the sparge needle was dropped into the same tube to ensure all the radioactivity was measured. The radioactivity present in all the four tubes was measured on the gamma counter to obtain the CPM values. These values were adjusted to the weight based on the net weight obtained above for both blood and tumor samples. Fractional [^11^C]CO_2_ (f(p,T),C11CO2) in the tubes was calculated with Eq. (5) using the following formula:

(5) f(p,T),C11CO2=CPMbase-CPMacidCPMbase

Because of difficulty in obtaining reliable estimates of tissue [^11^C]CO_2_ related to tissue loss during the rapid tissue homogenization, we performed additional studies of [^11^C]glutamine in HCC1806 and MCF-7 cell cultures, which was easily dissolved in acid without the need for homogenation, to confirm the fraction of anticipated tissue radioactivity in the form of [^11^C]CO_2_ using the acid/base method used for tissue. HCC1806 cells were grown in 6 cm dishes on DMEM with 10 % FBS and 4 mM glutamine, incubated in 5 % CO_2_. 370 MBq (10 mCi) of synthesized [^11^C]glutamine was added to growth media in an approximately 1:2 volume: volume ratio and split equally over 8 total confluent dishes and incubated for 30 min to align with imaging studies. Samples were then split into acid (n=4) and basic (n = 4) plates. They were processed and analyzed like tissue samples described in the *in vivo* [^11^C]CO_2_ studies.

### 2.8. Carbon-13 glutamine in vitro studies

To confirm metabolite identities and measured fractional concentrations for [^11^C]glutamine and downstream metabolites, we conducted a stable isotope tracing study in breast cancer cells using L-[5-^13^C]-glutamine ([^13^C]glutamine) at the metabolic core lab in Children’s Hospital of Philadelphia. HCC1806 and MCF-7 cells were incubated with 1μM CB-839 or vehicle (DMSO, 0.05 %) in DMEM containing 10 % FBS for 24 h. Afterwards, the culture media was replaced by glutamine-free DMEM containing 2 mM [^13^C]glutamine and 1μM CB-839 or vehicle (DMSO, 0.05 %) and cells were incubated for 30 min. After removing the media and washing with PBS twice, the cells were collected in 4 % perchloric acid followed by neutralization with KOH. Neutralized extracts were subjected to either AG-1 column (100–200 mesh, 0.5 × 2.5cm; Biorad) for enriching the organic acids, or AG-50 (100–200 mesh, 0.5 × 2.5cm; Biorad) for enriching amino acids. The collected samples were then converted to t-butyldimethylsilyl derivatives as described previously [24,25]. Measurement of ^13^C-isotopomer enrichment was performed on either an Agilent Triple Quad 6410 mass spectrometer combined with an Agilent LC 1290 Infinity or Hewlett-Packard 5971 MSD (mass selective detector), coupled with a 5890 HP-GC, GC–MS Agilent System (6890 GC/5973 MSD) or a Hewlett Packard HP-5970 MSD using electron impact ionization with an ionizing voltage of − 70 eV and an electron multiplier set to 2000 V. GC–MS measurement of ^13^C enrichment in glutamine metabolites including TCA cycle intermediates were performed as previously described [24,25]. The isotopic enrichment of [^13^C]glutamine isotopomers was monitored using ions at m/z431,432,433,434,435 and 436 for M0, M1, M2, M3, M4, and M5 (containing one to five ^13^C atoms above M0, the natural abundance), respectively. Likewise, isotopic enrichment in [^13^C]glutamate isotopomers was monitored using ions at m/z432,433,434,435,436, and 437 for M0, M1, M2, M3, M4, and M5, respectively, [^13^C]aspartate isotopomers at m/z418,419,420,421, and 422 (corresponding to M0, M1, M2, M3, and M4, respectively), [^13^C]malate isotopomers at m/z419,420,421,422, and 423 (M0, M1, M2, M3, and M4, respectively), [^13^C]fumarate isotopomers at m/z287,288,289,290, and 291 (M0, M1, M2, M3, and M4, respectively), ^13^C succinate isotopomers at m/z289,290,291,292, and 293 (M0, M1, M2, M3, and M4, respectively), and [^13^C]citrate isotopomers at m/z459,460,461,462,463,464, and 465 (M0, M1, M2, M3, M4, M5, and M6, respectively). All isotopomers of a specific ^13^C-labeled metabolite were summed.

### 2.9. Image derived, estimated metabolite-specific time-activity curves

Our overall approach used the image-derived total radioactivity curves for blood and tumor, the measured fractional activity in different tissue components (*e.g*., volatile fraction in the form of [^11^C]CO_2_, and non-volatile fraction separated into tumor pellet *versus* soluble fraction), as well as HPLC-based analysis of fractional activity of tissue metabolites in soluble fraction, to generate tissue type, and metabolite-specific time-activity curves for blood and tumors. Specifically, the fractional activity in ^11^C-labeled CO_2_ *versus* non-CO_2_, blood fractional activity in the red blood cells (RBC)’s *versus* plasma; tumor pellet (*versus* soluble fraction); and radiometabolite activity in the form of ^11^C-labeled glutamine, glutamate, or other metabolites, all of which we collected at 10, 20, and 30 min. For blood measures, namely image-derived %IA/g and fractional activity, were averaged for mice receiving either vehicle (HCC1806 VEH + MCF-7 VEH) or CB-839 (HCC1806 CB-839 and MCF-7 CB-839); noting that CB-839 can alter systemic glutamine and glutamate metabolism. For tumor measures, the mean and standard error of the mean of image-derived %IA/g and fractional activity measures for 4 groups are presented: HCC1806 VEH, HCC1806 CB-839, MCF-7 VEH, and MCF-7 CB-839.

We next generated plasma and tumor curves at the sampling interval provided by the image-based time-activity curves by interpolating the fractional measures of radioactivity in the form of [^11^C]glutamine (f(p;T),C11GLN), [^11^C]glutamate (f(p;T),C11GLU), ^11^C-other (f(T),C11OTH), [^11^C]CO_2_, (f(p,T),C11CO2) and ^11^C-labeled macromolecules (pellet). With t=0 starting conditions of the study all the radioactivity measured is from [^11^C]glutamine, and none in the form of [^11^C]glutamate, ^11^C-other, or [^11^C]CO_2_, with decreases in the fractional parent compound and increases in metabolites over time. To realistically represent the expected fractional activity as a function of time, from tracer injection to 30 min p.i., empiric functions were used to fit fractional data for blood and tissue metabolites- namely, double exponential, hill, single exponential and second-degree polynomial. The goodness-of-fit for each model was compared with sum of squared error metric, and curves were visually inspected to ensure that physiologic assumption of achieving steady state equilibrium concentration over time was maintained.

Based on this analysis, for the plasma curves, the single exponential curve was fit to the [^11^C]CO_2_ fraction prior to interpolation of the fractional data to match the temporal resolution of the image-derived total plasma curve. The [^11^C]CO_2_ signal (Cp,C11CO2) was then subtracted from total plasma curve ($C_{p,\text{Total}}$). To fit the activity of non-volatile metabolites in plasma, a single exponential function was chosen for fitting [^11^C]glutamine and [^11^C]glutamate. Then, metabolite-specific TACs for plasma (Cp,C11GLN and Cp,C11GLU), were generated by interpolating the fitted fractions and multiplying by the [^11^C]CO_2_-free plasma curve (Cp,C11CO2-free). The total plasma curve ($C_{p,\text{Total}}$) was partitioned into three input TACs to the model as shown in Eqs. (6)–(10). All units are in %IA/g. (See Supplemental Table 1 for full listing of definitions for the variables.)

(6) Cp,Total=Cp,C11GLN+Cp,C11GLU+Cp,C11CO2

(7) Cp,C11CO2=Cp,Total*fp,C11CO2

(8) Cp,C11CO2-free=Cp,Total-Cp,C11CO2

(9) Cp,C11GLN=Cp,C11CO2-free*fp,C11GLN

(10) Cp,C11GLU=Cp,[11C]CO2-free*fp,C11GLU

For the tumor time-activity curves, the tumor [^11^C]CO_2_ fraction (fT,C11CO2) was fit to a hill function prior to multiplication with the total tumor curve ($C_{T,\text{Total}}$) and the [^11^C]CO_2_ signal (CT,C11CO2) was subtracted from the total tumor curve. The [^11^C]CO_2_-free curve (CT,C11CO2-free) was separated based on fractional activity in supernatant (soluble) ($f_{T,\text{soluble}}$) and pellet ($1-f_{T,\text{soluble}}$). The soluble tissue extract curve was then estimated by multiplying the fitted soluble fraction with the [^11^C]CO_2_-free curve. Then, the tumor metabolite-specific fractions of the soluble tissue extract curve were estimated by fitting fractional [^11^C]glutamine (assumed [^11^C]glutamine at t=0min is 1) and [^11^C]glutamate fractional data to hill and single exponential model, respectively. The other metabolite fraction was estimated by subtracting the glutamine and glutamate fraction from 1fT,C11OTH. Each of the fractions were interpolated and multiplied by the tissue soluble activity TAC (Supplemental Fig. 2). This resulted in total soluble tissue extract curve ($C_{T,\text{soluble}}$), pellet activity curve, and metabolite-specific time activity curves (CT,C11GLN,CT,C11GLUand CT,C11OTH) seen in Eqs. (11)–(19) for the tumor, in additional to the total tumor signal ($C_{T,\text{Total}}$). All units are in %IA/g:

(11) CT,Total=CT,C11CO2+CT,pellet+CT,soluble

(12) CT,C11CO2=CT,Total*fT,C11CO2

(13) CT,C11CO2-free=CT,Total-CT,C11CO2

(14) CT,soluble=CT,C11CO2-free*fT,soluble

(15) CT,soluble=CT,C11GLN+CT,C11GLU+CT,C11OTH

(16) CT,C11GLN=CT,soluble*fT,C11GLN

(17) CT,C11GLU=CT,soluble*fT,C11GLU

(18) fT,C11OTH=1-fT,C11GLN-fT,C11GLN

(19) CT,C11OTH=CT,soluble*fT,C11OTH

To temporally align the blood and tissue curves to account for delay from the heart to the tumor, the tumor time activity curves ($C_{T,\text{Total}}$) for each mouse were delay corrected to align initial rise of the blood clearance (10 % of peak activity) to the tissue curve [22].

### 2.10. Statistical analysis

Data are presented as mean ± standard error of mean (SEM) (presented with error bars). All statistical analysis was performed in Excel and GraphPad Prism. For comparing time activity curves a paired 2-sample, 2-tailed t-test was used at common time points. For comparisons of metabolite specific % radioactivity of HPLC data, unpaired one-tailed t-tests were performed to evaluate differences between groups, given expected unidirectional changes in blood and tumor tissue of glutamine (increase) and glutamate (decrease) upon CB-839 administration. For both types of tests, α was set at 0.05.

## 3. Results

### 3.1. Image-derived, whole blood clearance and tumor curves

The whole blood time-activity curves were similar, regardless of tumor model (HCC1806 or MCF-7) or if the mouse was treated with CB-839 or vehicle (Fig. 1A). Total tumor signal curves when comparing all post-treatment mice cohorts do not demonstrate any significant differences or trends for HCC1806 *vs* MCF-7, or for vehicle *vs* CB-839 for either tumor model (Fig. 1B), demonstrating significant overlap between the curves of each tumor type and treatment condition. A statistically insignificant trend of higher tumor TACs in the MCF-7 vehicle is seen, partially related to higher tumor glutamine pool sizes in the less glutaminolytic MCF-7 relative to HCC1806 and concordant with the higher uptake of the non-metabolized [^18^F]fluoroglutamine seen in MCF-7 relative to HCC1806 in this model [12]. As noted in the Discussion section the slightly larger pool size in the vehicle *versus* CB-839 cells may reflect the imaging of glutamine synthase which recycles glutamate to glutamine in more differentiated cancer cells such as ER+ breast cancer [26]. Representative images of mice in each condition are provided in Supplemental Fig. 3. Unlike our prior studies using the non-metabolized [^18^F]fluoroglutamine in the same animal models [12], the total radioactivity T/B derived from [^11^C]glutamine PET does not show significant differences in CB-839 *vs*. vehicle treated glutaminolytic HCC1806 tumors and was therefore, in the absence of metabolite correction, unable to provide a robust indicator of inhibition of glutaminase activity in highly glutaminolytic tumors (Fig. 1C–D). However, when comparing the interval change in T/B between pre-treatment and post-treatment imaged mice, a borderline statistically significant trend was observed between MCF-7 vehicle treated mice and MCF-7 CB-839 treated mice, with decrease in T/B upon CB-839 treatment (Fig. 1D).

To assure comparability of tumor and blood image-derived time-activity curves, we compared T/B obtained for image-derived *versus* tissue samples and counted measures at 10, 20, and 30 min. These values showed a prevalence towards higher T/B ratios for tissue samples over image-derived samples (Supplemental Fig. 4). The lower image-derived T/B ratios may be due to signal loss from partial volume effects in the tumor, especially considering the prevalence of necrosis in the aggressive HCC1806 cell line. Mouse tumor %IA/g was estimated using a “peak” region around a non-necrotic portion of the tumors (Fig. 1B), which was also the tissue used for well counting. While the partial volume effects may still impact the relationship between the tumor curves and blood curves, it does not impact the relative profiles of metabolites for the blood and tissue curves.

### 3.2. Radioactivity extraction – partition of blood into RBCs vs plasma and in both blood and tumor into supernatant versus pellet

In the blood of mice bearing HCC1806 or MCF-7 tumor and regardless of CB-839 or vehicle treatment, the radioactivity partitions slightly higher in plasma *versus* red blood cells (RBCs) throughout the 30-minute experimental time course, indicating that the radio-metabolites were freely exchanged between plasma and RBC, and there was no sequestration in RBCs. In both plasma and tumor samples, the majority of radioactivity was present in the supernatant *versus* the pellet (Supplemental Fig. 5), of similar magnitude to prior studies with [^18^F] fluoroglutamine [11,12], suggesting only a small amount of parent tracer and/or metabolite incorporation in macromolecules out to 30 min p.i. and minimal, if any sequestration of radioactivity in the blood from the plasma fraction.

### 3.3. Analysis of non-volatile labeled metabolites in blood and tumor tissue

**Fig. 2.**
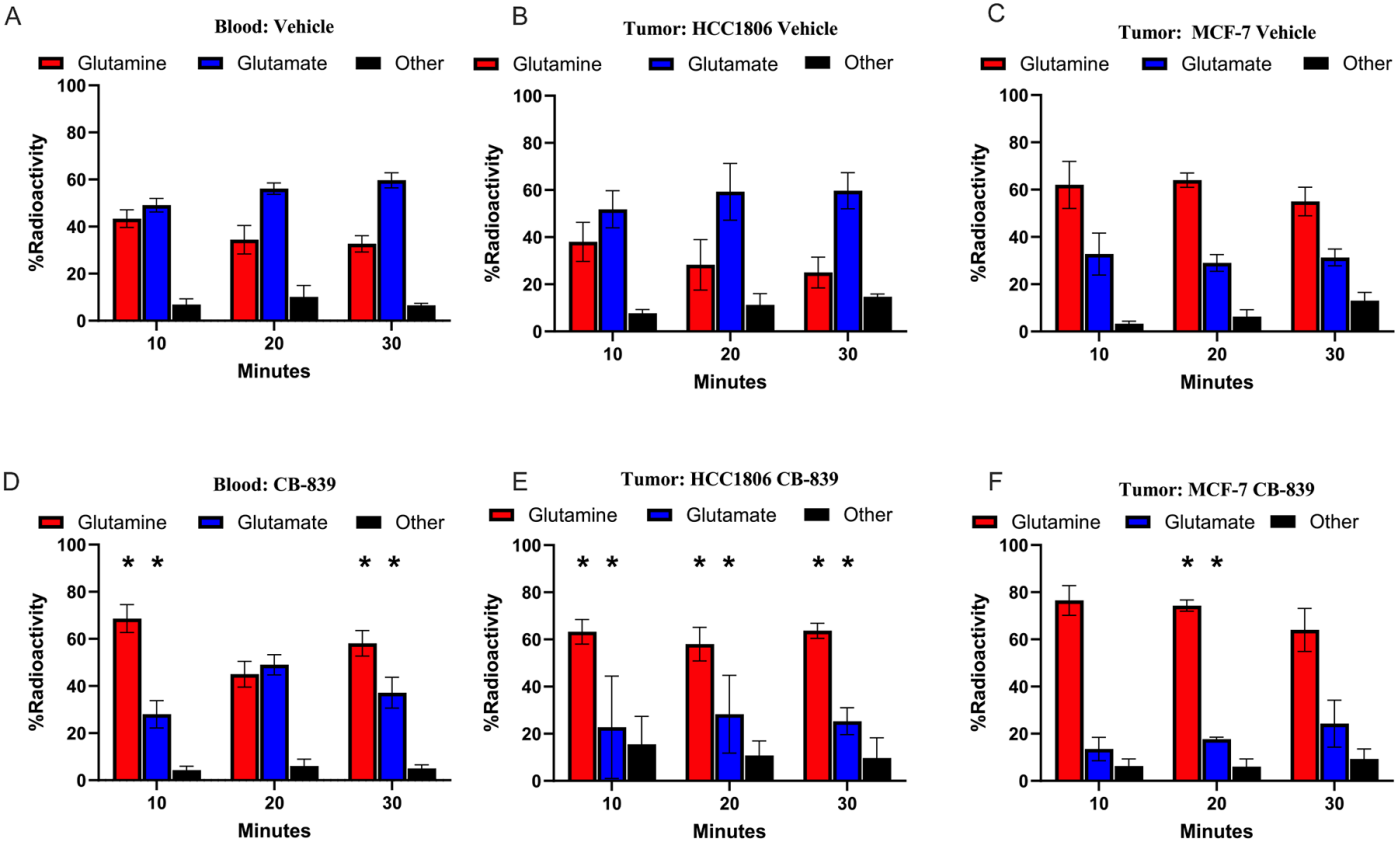
Metabolite-specific % Radioactivity HPLC data of extracted tissue from TNBC (HCC1806) and HR+ (MCF-7) breast cancer cell line flank xeongraft-bearing mice administered L-[5-^11^C]glutamine via tail vein injection (n > 3): A) Vehicle treated blood B) HCC1806 Vehicle treated tumor C) MCF-7 Vehicle treated tumor D) CB-839 treated blood E) HCC1806 CB-839 treated tumor F) MCF-7 CB-839 treated tumor. For each graph, each timepoint consists of n = 3–11 biological replicates. Values are plotted as averages with error bars representing ±standard error of the mean. *p < 0.05, via unpaired t-test analysis (1 tailed, unequal variance) comparing CB-839 metabolites (D–F) to matched timepoint Vehicle metabolites (A–C).

Between 0.85 and 0.9 of the total non-volatile metabolite activity fraction in plasma and tumor supernatant consists of [^11^C]glutamine and [^11^C]glutamate, irrespective of tumor genetic background (Fig. 2). As expected, CB-839 inhibits some systemic metabolism of [^11^C]glutamine, resulting in a higher fraction of [^11^C]glutamine and lower fraction of [^11^C]glutamate in the plasma of CB-839 animals for both tumor models (Fig. 2A *versus* D). In HCC1806 tumors, the relative contribution of [^11^C] glutamine *versus* [^11^C]glutamate to overall signal is reversed after CB-839 compared to vehicle treatment (Fig. 2B *versus* E), consistent with increased glutamine and decreased glutamate concentration in response to GLS inhibition in glutaminolytic tumors. However, this is not the case for MCF-7, where [^11^C]glutamine is the dominant radioactive species in both vehicle and CB-839-treated animals (Fig. 2C *versus* F) out to 30 min. This was anticipated due to the low-level of GLS in MCF-7 compared with HCC1806 tumors.

### 3.4. Carbon-13 glutamine in vitro studies

To support the results observed in [^11^C]glutamine PET and metabolite studies, cell culture experiments were performed with L-5-[^13^C]glutamine ([^13^C]glutamine), which is labeled at the same position (carbon-5) as the ^11^C PET tracer. The soluble small molecule metabolites at 30 min after incubation with [^13^C]glutamine are summarized for HCC1806 and MCF-7 cells (Supplemental Fig. 6). Like [^11^C]glutamine *in vivo*, the predominant soluble metabolites in both cell lines with administered [^13^C]glutamine *in vitro* are [^13^C]glutamine and [^13^C] glutamate, whereas only minute levels of other metabolites were detected, specifically TCA intermediates ([^13^C]citrate, [^13^C]malate, [^13^C]aspartate and [^13^C]fumarate). In HCC1806 cells, CB-839 treatment reduced the cellular GLU/GLN ratio from ~1:1 to approximately 1:6 and decreased the labeling of TCA cycle intermediates compared to vehicle treatment. It is expected that changes in [^13^C]glutamine and [^13^C] glutamate in HCC1806 cells *in vitro* accounted for most of the change measured in [^11^C]glutamine and [^11^C]glutamate in HCC1806 tumors *in vivo*. These findings confirm the mouse model [^11^C]glutamine data suggesting retention of GLS-metabolized [^11^C]glutamine as the major source of the large intracellular [^11^C]glutamate pool in glutaminolytic TNBC cells treated with vehicle, with the majority of the [^11^C]glutamate intracellular pool signal shifting to [^11^C]glutamine signal upon glutaminase inhibition.

### 3.5. [^11^C]CO_2_ estimation

Besides non-volatile metabolites of [^11^C]glutamine, we also attempted to measure the volatile metabolites, especially [^11^C]CO_2_, which represents the end product of [^11^C]glutamine metabolism through the glutaminolysis pathway, where L-5-[^11^C]-glutamate ([^11^C] glutamate) is converted to L-5-[^11^C]-alpha-ketoglutarate ([^11^C]alpha-ketoglutarate), which is further metabolized through the TCA cycle to produce [^11^C]CO_2_. In general, the [^11^C]CO_2_ activity fraction of the total ^11^C signal ranged from 0.1 and 0.35, for any condition, for both blood clearance curves and tumors, without clear trend and with significant variability, likely secondary to the complication of estimating volatile compounds in tissue samples (Supplemental Table 2).

*In vitro*, [^11^C]CO_2_ contribution to HCC1806 cells in culture incubated with [^11^C]glutamine over a period of 30 min demonstrated a lower contribution to overall signal compared to *in vivo* data, contributing to an approximate fraction of 0.15 of the total signal compared to the approximately 0.3 observed *in vivo* (Supplemental Fig. 7), confirming a low-fraction of total radioactivity present as [^11^C]CO_2_, and comparable to the fractional blood [^11^C]CO_2_ radioactivity.

### 3.6. Estimated metabolite-specific time-activity curves

**Fig. 3.**
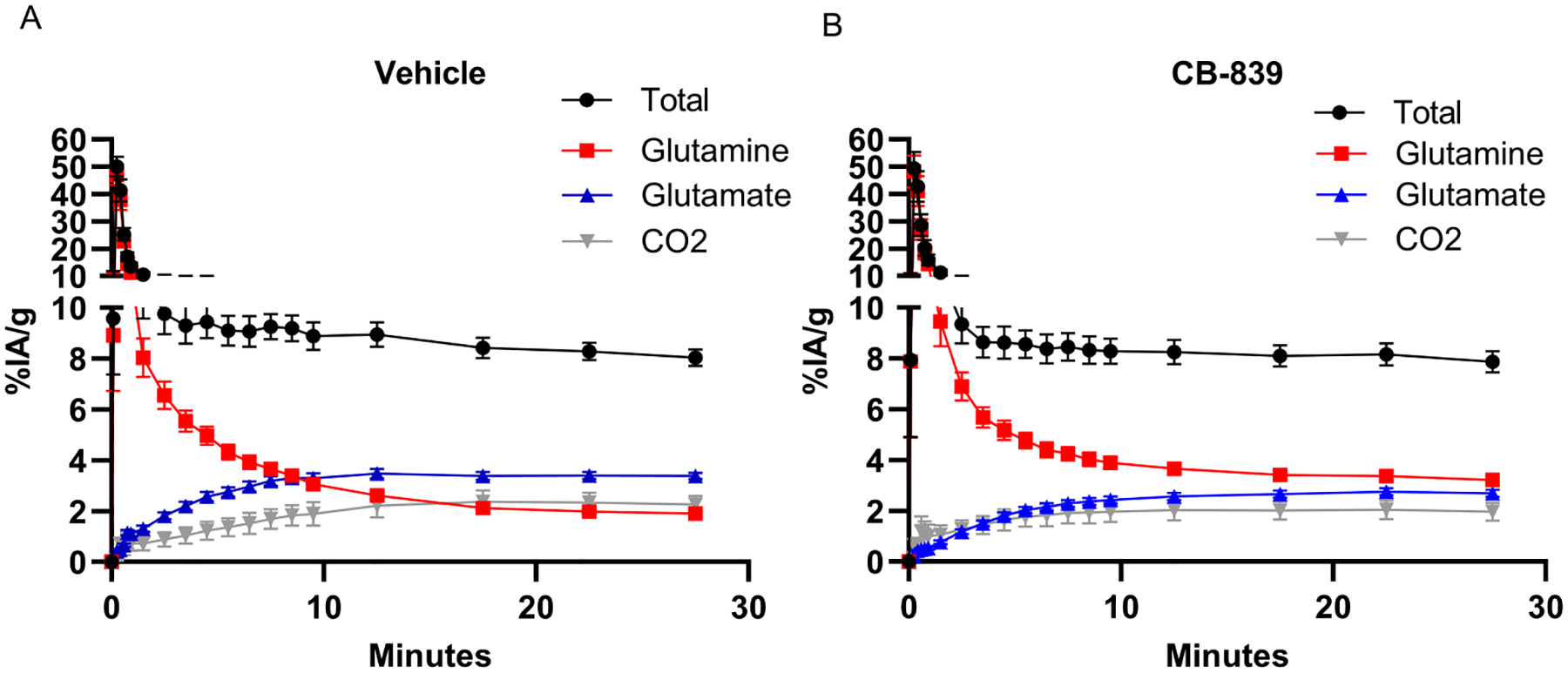
Metabolite-specific blood clearance curves across conditions of extracted tissue from TNBC (HCC1806) and HR+ (MCF-7) breast cancer cell line flank xenograft-bearing mice administered L-[5-^11^C]glutamine via tail vein injection. A) Blood clearance curves for vehicle (n = 17). B) Blood clearance curves for CB-839 (n = 12). Values are plotted as averages with error bars representing ±standard error of the mean.

For the blood, for both tumor models and in either CB-839 or vehicle treatment, a similar phenotype is observed. Throughout the 30 min time course, the contribution of [^11^C]glutamine to total ^11^C signal decreases, while the contribution of [^11^C]glutamate to total ^11^C signal increases at a somewhat faster rate for vehicle treated animals compared to CB-839 animals, as expected (Fig. 3), consistent with systemic GLS inhibition. In the vehicle treated mice, the [^11^C]glutamine and [^11^C]glutamate curves cross between 10 and 15 min, with the majority of total ^11^C signal comprised of [^11^C]glutamate at the end of the 30-minute imaging experiment. However, in the CB-839 treatments, the curves do not cross and the amounts of contribution of [^11^C]glutamine and [^11^C]glutamate are approximately equal at the end of the 30-minute imaging experiment.

**Fig. 4.**
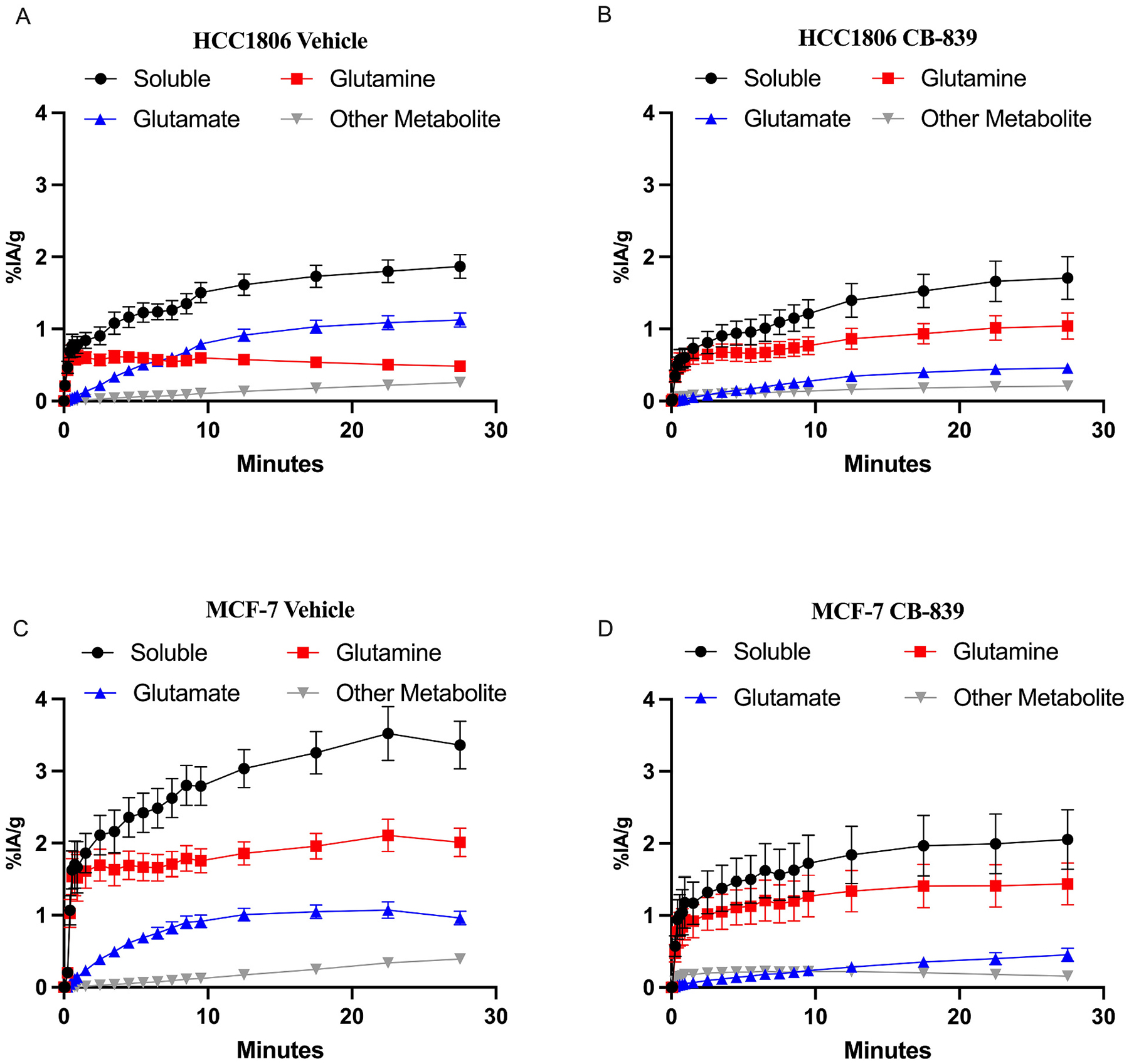
Tumor soluble signal curves partitioned into individual metabolites of breast cancer cell line flank xenograft-bearing mice administered a bolus of L-[5-^11^C] glutamine via tail vein injection. A) HCC1806 Vehicle (n = 9). B) HCC CB-839 (n = 7). C) MCF-7 Vehicle (n = 9). D) MCF-7 CB-839 (n = 7). Values are plotted as averages with error bars representing ±standard error of the mean.

A similar analysis can be applied to obtain the estimated tumor soluble signal, by combining the tumor signal time course partitioning into soluble and pellet fractions (Supplemental Fig. 5E–F) to the total tumor time activity curves (Supplemental Fig. 8). From the soluble tissue extract curve, the inclusion of tumor metabolite analysis from Fig. 2 and using Eqs. (12)–(15) revealed notable differences in [^11^C]glutamine and [^11^C]glutamate time course of HCC1806 in response to vehicle *vs* CB-839 treatment: while [^11^C]glutamate rises and crosses [^11^C]glutamine curve in vehicle tumors, [^11^C]glutamate remains much lower than [^11^C]glutamine during the entire time course in CB-839 treated tumors (Fig. 4A–B). In contrast, in MCF-7 mice treated with vehicle, the metabolite-specific TACs exhibit a similar kinetic appearance to the CB-839 treated tumors (Fig. 4C–D) and similar TACs to CB839-treated HCC1806 tumors, as expected (comparing Fig. 4C–D with Fig. 4B). Upon CB-839 treatment in MCF-7 mice, the fractional contribution of [^11^C]glutamate to total tumor signal is the smallest of all conditions (Fig. 4D).

## 4. Discussion

Studies in cells and mouse models using both [^11^C]glutamine and [^13^C]glutamine provide insights into the kinetics of glutamine metabolism and the impact of GLS antagonists in glutaminolytic breast cancer tumor models. The observed rapid metabolism of [^11^C]glutamine to [^11^C]glutamate in the blood and tumors of vehicle treated TNBC tumor-bearing mice (HCC1806) is in accordance with GLS mediated conversion of glutamine to glutamate. Conversely, in CB-839 treated mice, plasma and tumor analysis showed that the parent [^11^C]glutamine maintains a higher proportion of overall signal; this is an expected result as CB-839 inhibits GLS activity in all tissues expressing *GLS1*, slowing kinetics of [^11^C]glutamate formation. The finding that the proportion of [^11^C] glutamate and “other” downstream [^11^C]metabolites are lower, but not eliminated, in the blood in the presence of CB-839, is expected. CB-839 inhibits the kidney-type gene isoforms of *GLS1* (KAG and GAC) but does not inhibit the liver-type gene isoform of *GLS2*, which is highly expressed in liver and other organs and accounts for a substantial portion of total body GLS activity [8,15].

The tumor metabolite profiles observed in estrogen-receptor positive (ER+) tumor bearing mice (MCF-7) were distinct from the TNBC tumor-bearing mice (HCC1806) as expected given low baseline GLS activity in hormone-receptor positive (HR+) cells. Vehicle treated MCF-7 tumors have a profile similar to TNBC tumors treated with CB-839, in keeping with their low intrinsic GLS activity. The metabolite-specific measurements showed clear differences for GLS-high (HCC1806) *versus* GLS-low (MCF-7) tumors pre- and post-CB-839. However, most of the activity in both tumor types was in the form of either [^11^C]glutamine or [^11^C] glutamate, and, as such, the total tumor activity and total activity tumor/blood ratio change from pre- and post-GLS inhibition conditions demonstrated only modest changes (Fig. 1B–C). Furthermore, the relatively small fraction of total ^11^C signal from [^11^C]CO_2_ derived from the tumor cells *in vitro* compared to *in vivo* studies supports the concept that a large proportion of [^11^C]CO_2_ signal observed in tumor xenografts is likely secondary to free exchange with plasma [^11^C]CO_2_ from systemic metabolism. As such, measuring total tumor radioactivity by PET after L-5-[^11^C]-glutamine administration may pose challenges for inferring GLS activity and inhibition without both blood and tumor metabolite analysis, the latter of which cannot be done in humans. A non-metabolized glutamine ([^18^F]fluoroglutamine) or similarly processed amino-acid analogue ([^18^F]Fluciclovine) may be a more robust strategy for assessing GLS activity and inhibition in patients, given the significant confounding effects of both competing signal changes of [^11^C]glutamine and [^11^C] glutamate, and significant noise from [^11^C]CO_2_, as mentioned above [12,13,27]. An alternative approach utilizing a labeled carbon tracer has been demonstrated with hyperpolarized MRI, in which both glutamine and glutamate can be monitored noninvasively in a single acquisition [28].

While our studies indicated the practical complexity of interpreting imaging data derived from [^11^C]glutamine PET, our results provide some insights into the biochemical kinetics of glutaminolytic cancers. In general, findings from the tracer analysis estimations demonstrate high concordance with previously obtained observations from direct experimentation in this model [12,29]. First, the estimated increase of approximately 2-fold retention of [^11^C]glutamine seen in HCC1806 upon glutaminase inhibition is congruent with the approximately 4-fold increase in native glutamine pool size assayed by *ex vivo* ^1^H MR CEST in this model previously upon CB-839 treatment [12]. This may limit the efficacy of GLS inhibition in decreasing flux through GLS, as increasing cellular glutamine levels will continue to drive the reaction even with substantial glutaminase inhibition. In the absence of GLS inhibition, the soluble [^11^C]glutamine signal is greater in MCF-7 than HCC1806. Furthermore, this MCF-7 [^11^C]glutamine signal does not significantly increase upon CB-839 treatment. These trends were previously observed with *ex vivo* ^1^H MRS as well [12]. Similarly, estimated [^11^C]glutamate signal at 30 min post-injection decreases in HCC1806 tumors in the presence of CB-839 to 40 % of vehicle-treated mice, similar to the decrease in glutamate pool size observed by *ex vivo* ^1^H MRS to 33 % of vehicle-treated mice (Fig. 4) [29].

An interesting finding, meriting further study is significant cellular retention of label in the form of [^11^C]glutamate in a large cellular glutamate pool confirmed analysis by mass spectrometry from cell culture samples using [^13^C]glutamine and also seen in prior *in vivo* studies using MR CEST [29]. This implies that the large glutamate pool in untreated HCC1806 cells is derived from catabolism of glutamine and not from circulating glutamate. This indirectly suggests that extracellular glutamate and intracellular glutamate pools have incomplete communication, as has been noted in other studies [30–32] and raises interesting questions on the subcellular localization of the glutamate pool meriting further study. Of note, there is borderline higher total radioactivity and [^11^C]glutamine activity for vehicle treated *versus* CB-839 treated MCF-7 cells (Fig. 1b, Fig. 4c–d) that may be related to “metabolic recycling” by conversion of intracellular glutamate to glutamine by glutamine synthase, which may contribute to high levels of glutamine observed in ER+ cells [26]; this is also a topic meriting further study.

The limitations of the current study are as follows: First, the study utilizes two representative breast cancer xenograft models, one has high GLS activity (HCC1806) and another low (MCF-7). As such, the conclusions of this study could be bolstered by expanding results to a greater number of cell lines in each group. Second, there are inherent experimental challenges to measuring complex blood and tissue metabolite profiles using of [^11^C]glutamine given the short physical half-life of ^11^C (20.4 min). To address this, we utilized *in vitro* studies of breast cancer cell lines treated with stable ^13^C isotope-labeled [^13^C]glutamine to confirm our significant observations. We note that the use of *in vitro* ^13^C-Glutamine only studies to validate the [^11^C]glutamine results is a limitation of our study. Utilizing previously published methodology, we are currently pursuing [^13^C]glutamine infusions *in vivo* in this model [33]. Third, these studies did not directly address the degree of tumor metabolite signal from uptake of plasma metabolites, specifically, tumor uptake of [^11^C]glutamate from the plasma *versus* intratumoral conversion of [^11^C]glutamine to [^11^C]glutamate. However, the later time-point [^11^C]glutamate/[^11^C]glutamine ratio in untreated HCC1806 tumors is higher than the in the blood, yet is lower in CB-839 treated tumors than blood, suggesting that changes in tumor [^11^C]glutamate are not simply explained by the transport of [^11^C]glutamate in the blood arising from systemic metabolism Additionally, *in vitro*, highly glutaminolytic TNBC demonstrated findings consistent with the proposed *in vivo* [^11^C]glutamine model, in further support of an *in vivo* model that focuses predominantly on [^11^C]glutamine uptake and metabolism. We note that these are early “plausibility” studies of a proposed kinetic model based on limited mouse data, generating hypotheses needing further mechanistic validation. Additionally, kinetic analysis of the data is an ongoing future direction which is currently being conducted. The largely empiric interpolation scheme implemented here will be further studied in a kinetic modelling study. Furthermore, we are pursuing the interplay of glutaminolysis and the xCT cystine/glutamate antiporter, as recently published, and how this interplay affects the uptake of PET radiotracers for these pathways as reflected by a recent publication from our group [34].

### 4.1. Conclusion

Time activity curves of the blood and tumor derived from [^11^C] glutamine PET appear similar in mice bearing either HCC1806 or MCF7 tumors treated by CB-839 or vehicle. Metabolite analysis showed the rapid metabolism of [^11^C]glutamine to [^11^C]glutamate that is retained in a large intracellular pool resulting in overlap of total time activity curves for glutaminolytic (HCC1806) *versus* non-glutaminolytic (MCF-7) tumor models. This overlap poses challenges for inferring GLS activity *in vivo* from [^11^C]glutamine PET total radioactivity measurements. This suggest limited utility for L-5-[^11^C]-glutamine PET for inferring tumor GLS activity and its specific antagonism by drug inhibitors, which may be better performed using non-metabolized glutamine analogs. Our observations, however, do support prior biochemical findings in glutaminolytic tumors and indicate that, in the TNBC cell line tested, much of the glutamate generate by GLS is retained in a large intracellular pool. Future studies should further assess the nature of the cellular glutamate pool derived from glutaminolysis to better target glutamine metabolism in breast cancer and other cancers.

## Supplementary Material

1

## KEY POINTS QUESTION:

Whether or not L-5-[^11^C]-glutamine ([^11^C] glutamine or [^11^C]GLN) PET is able to detect inhibition of glutaminase in human triple negative breast cancer xenografts that are dependent on glutamine metabolism and have high glutaminase activity.

## PERTINENT FINDINGS:

Utilizing xenograft mouse models of human breast cancer, cohorts of mice were injected with [^11^C]glutamine and underwent respectively dynamic PET imaging and were euthanized for metabolites analyses after treatment of a glutaminase inhibitor CB-839 or vehicle. Kinetic analysis of dynamic [^11^C]glutamine PET time-activity curves combined with *ex vivo* metabolites analyses of the blood and tumor enabled estimation of [^11^C]glutamine metabolism through glutaminase to be distinguished in CB-893 *versus* vehicle treated tumors.

## IMPLICATIONS FOR PATIENT CARE:

Although the use of a biochemically authentic glutamine PET tracer ([^11^C]glutamine) yields biologic insights, retention of ^11^C-activity in L-5-[^11^C]-glutamate ([^11^C] glutamate or [^11^C]GLU)in tumors with high GLS activity confounds the interpretation of non-invasive PET imaging studies, supporting the use of non-metabolized glutamine analogs such as 4-^18^F-(2*S*,4*R*)-ﬂuoroglutamine ([^18^F]ﬂuoroglutamine) as pharmacodynamic markers of GLS inhibition.

## Appendix A. Supplementary data

Supplementary data to this article can be found online at https://doi.org/10.1016/j.nucmedbio.2025.109092.
