# Supplementary material for: L-5-[11C]-glutamine PET of breast cancer: Preclinical studies in mouse models
Source: Nucl Med Biol. Author manuscript; Available in PMC 2026 Apr 12. (PMC13070223; doi:10.1016/j.nucmedbio.2025.109092)
Supplement: 1 [file NIHMS2162697-supplement-1.docx]

**SUPPLEMENTAL RESULTS**

| Abbreviations | Description | units |
| --- | --- | --- |
| $\boldsymbol{ƒ}_{\left( \boldsymbol{p}\boldsymbol{;}\boldsymbol{T} \right)\mathbf{,}\mathbf{[}^{\mathbf{11}}\mathbf{C}\mathbf{]}\boldsymbol{GLN}}$ | Fractional activity of [^11^C]glutamine in plasma/tumor | unitless |
| $\boldsymbol{ƒ}_{\left( \boldsymbol{p}\boldsymbol{;}\boldsymbol{T} \right)\mathbf{,}\mathbf{[}^{\mathbf{11}}\mathbf{C}\mathbf{]}\boldsymbol{GLU}}$ | Fractional activity of [^11^C]glutamate activity in plasma/tumor | unitless |
| $\boldsymbol{ƒ}_{\left( \boldsymbol{p}\boldsymbol{;}\boldsymbol{T} \right)\mathbf{,}\mathbf{[}^{\mathbf{11}}\mathbf{C}\mathbf{]}\boldsymbol{OTH}}$ | Fractional activity of [^11^C]other metabolites downstream of glutamate in plasma/tumor | unitless |
| $\mathbf{ƒ}_{\left( \boldsymbol{p}\boldsymbol{,}\boldsymbol{T} \right)\mathbf{,}\mathbf{[}^{\mathbf{11}}\mathbf{C}\mathbf{]}\mathbf{CO}_{\boldsymbol{2}}}$ | Fractional activity of [^11^C]CO_2_ as glutamate enters TCA cycle in plasma/tumor | unitless |
| $\boldsymbol{C}_{\boldsymbol{p}\boldsymbol{,}\boldsymbol{Total}}$ | Total activity in plasma | %ID/g |
| $\boldsymbol{C}_{\boldsymbol{p}\boldsymbol{,}\mathbf{[}^{\mathbf{11}}\mathbf{C}\mathbf{]}\boldsymbol{GLN}}$ | [^11^C]glutamine concentration in plasma | %ID/g |
| $\boldsymbol{C}_{\boldsymbol{p}\boldsymbol{,}\mathbf{[}^{\mathbf{11}}\mathbf{C}\mathbf{]}\boldsymbol{GLU}}$ | [^11^C]glutamate concentration in plasma | %ID/g |
| $\boldsymbol{C}_{\boldsymbol{p}\boldsymbol{,}\mathbf{[}^{\mathbf{11}}\mathbf{C}\mathbf{]}\boldsymbol{CO}_{\boldsymbol{2}}}$ | [^11^C]CO_2_ concentration in plasma | %ID/g |
| $\boldsymbol{C}_{\boldsymbol{T}\boldsymbol{,}\boldsymbol{Total}}$ | Total activity in tumor | %ID/g |
| $\boldsymbol{C}_{\boldsymbol{T}\boldsymbol{,}\mathbf{soluble}}$ | Concentration of tissue soluble extract | %ID/g |
| $\boldsymbol{C}_{\boldsymbol{T}\boldsymbol{,}\mathbf{[}^{\mathbf{11}}\mathbf{C}\mathbf{]}\boldsymbol{CO}_{\boldsymbol{2}}}$ | Concentration of [^11^C]CO_2_ in tumor | %ID/g |
| $\boldsymbol{C}_{\boldsymbol{T}\boldsymbol{,}\mathbf{[}^{\mathbf{11}}\mathbf{C}\mathbf{]}\mathbf{GLN}}$ | Concentration of [^11^C]glutamine in tumor | %ID/g |
| $\boldsymbol{C}_{\boldsymbol{T}\boldsymbol{,}\mathbf{[}^{\mathbf{11}}\mathbf{C}\mathbf{]}\mathbf{GLU}}$ | Concentration of [^11^C]glutamate in tumor | %ID/g |
| $\boldsymbol{C}_{\boldsymbol{T}\boldsymbol{,}\mathbf{[}^{\mathbf{11}}\mathbf{C}\mathbf{]}\mathbf{OTH}}$ | Concentration of [^11^C]other metabolites downstream of glutamate in tumor | %ID/g |

**Supplementary Table 1: Abbreviations and their descriptions**

| Tissue | Xenograft | Treatment | 10 min | 20 min | 30 min |  |
| --- | --- | --- | --- | --- | --- | --- |
| Blood | HCC1806 | Vehicle | 37 +/- 11 | 17 +/- 8 | 29 +/- 5 |  |
|  |  | CB-839 | 21 +/- 7 | 24 +/- 2 | 27 +/- 4 |  |
|  | MCF-7 | Vehicle | 27 +/- 4 | 21 +/- 3 | 23 +/- 7 |  |
|  |  | CB-839 | 27 +/- 2 | 25 +/- 7 | 24 +/- 5 |  |
| Tumor | HCC1806 | Vehicle | 36 +/- 16 | 18 +/- 7^1^ | 35 +/- 16^1^ |  |
|  |  | CB-839 | 45 +/- 14 | 58 +/- 20^1^ | 28 +/- 17^1^ |  |
|  | MCF-7 | Vehicle | 2 +/- 2^1^ | ^-^ | 11 +/- 0^2^ |  |
|  |  | CB-839 | 15 +/- 2 | 30 +/- 1 | 29 +/- 10 |  |
| **Supplementary Table 2. [^11^C]Glutamine metabolism: ^11^CO2 estimation (%±SEM) in the blood and tumor (n≥3, ^1^n=2, ^2^n=1 , ^-^n=0).** | | | | | |  |
|  |  |  |  |  |  |  |


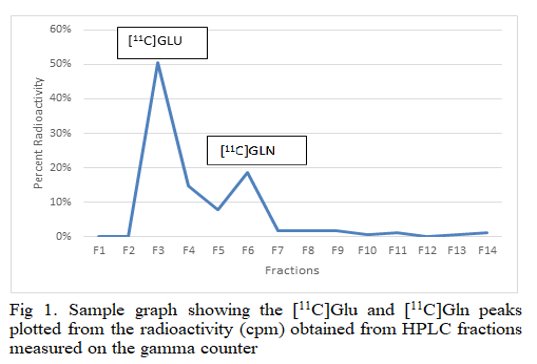


**Supplemental Figure 1:** Sample graph showing the [^11^C]glutamate ([^11^C]GLU) and [^11^C]glutamine ([^11^C]GLN) peaks plotted from the radioactivity (cpm) obtained from HPLC fractions measured on the gamma counter.


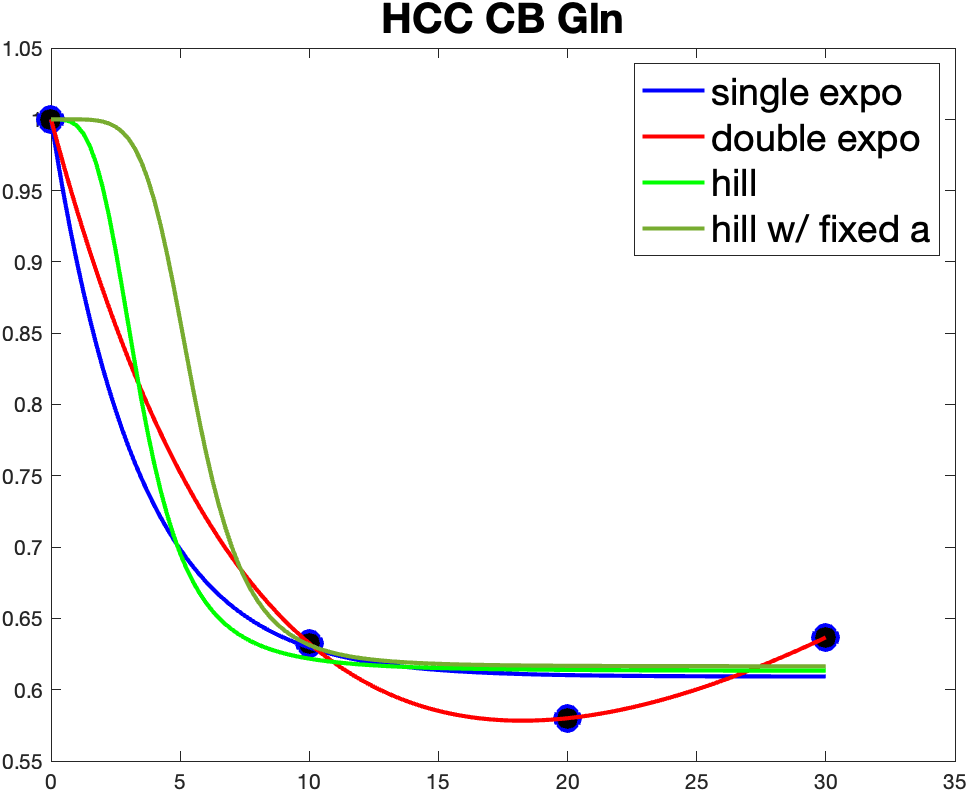


**Supplemental Figure 2:** Example of empiric interpolation methods compared for the fraction of total tumor curve are present as specific metabolites as function of time. **A**) Use of average values for each timepoint (t = 10 minutes, 20 minutes, 30 minutes), of [5-^11^C]GLN fraction to serve as points of estimate to create a curve-fit function that provides an estimated function of fraction of total signal that is [5-^11^C]GLN, per unit time, using the value of 1 at t = 0 minutes, meaning that the assumption is used that at the origin of the experiment the total signal is entirely [5-^11^C]GLN. The data was fit to *hill function*. **B-C**) Use of average values for each timepoint (t = 10 minutes, 20 minutes, 30 minutes), of [^11^C]GLU fraction and [^11^C]CO_2_ fraction that were fit to *single exponential* and *hill function*, respectively. This created metabolite-specific time activity curve of total signal for [5-^11^C]GLU and [5-^11^C]CO_2_, per unit time, using the value of 0 at t = 0 minutes, meaning that the assumption is used that at the origin of the experiment no [5-^11^C]GLU or is present.

B

A


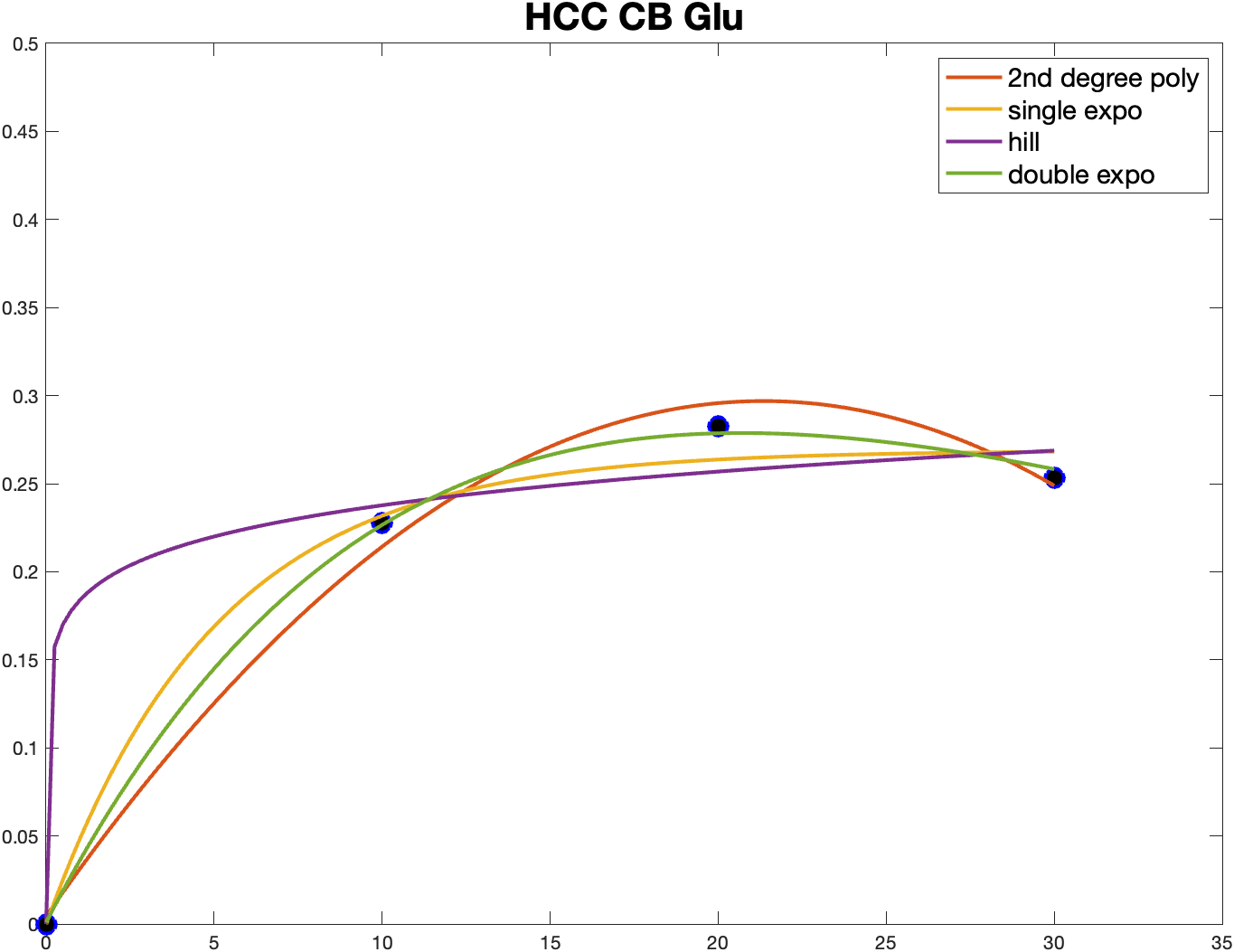


C


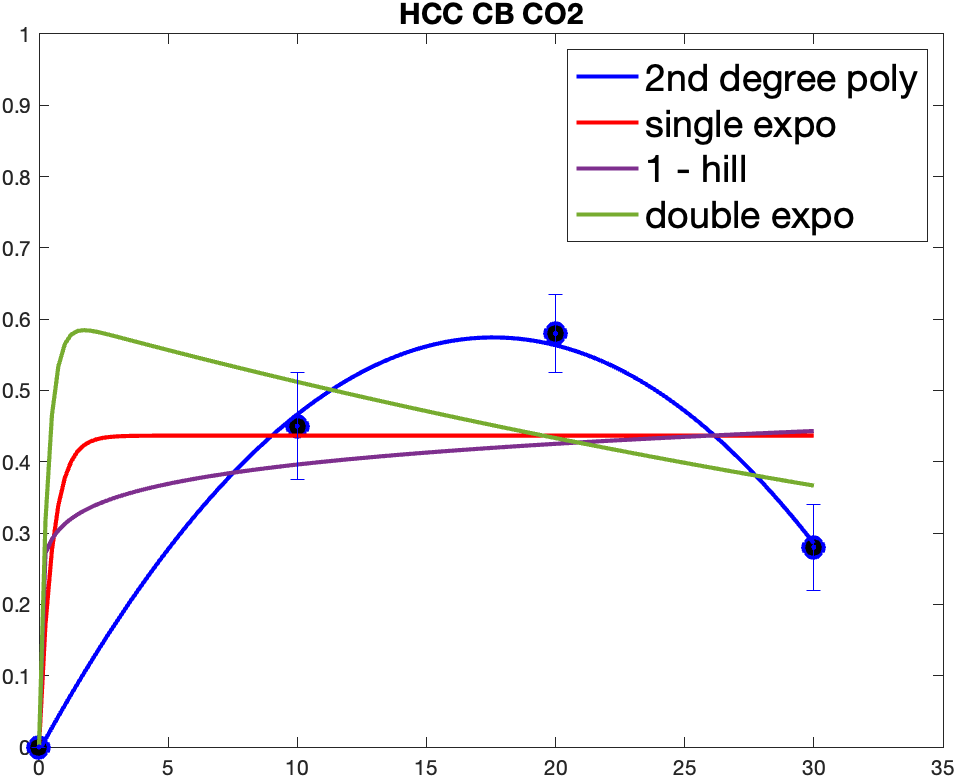


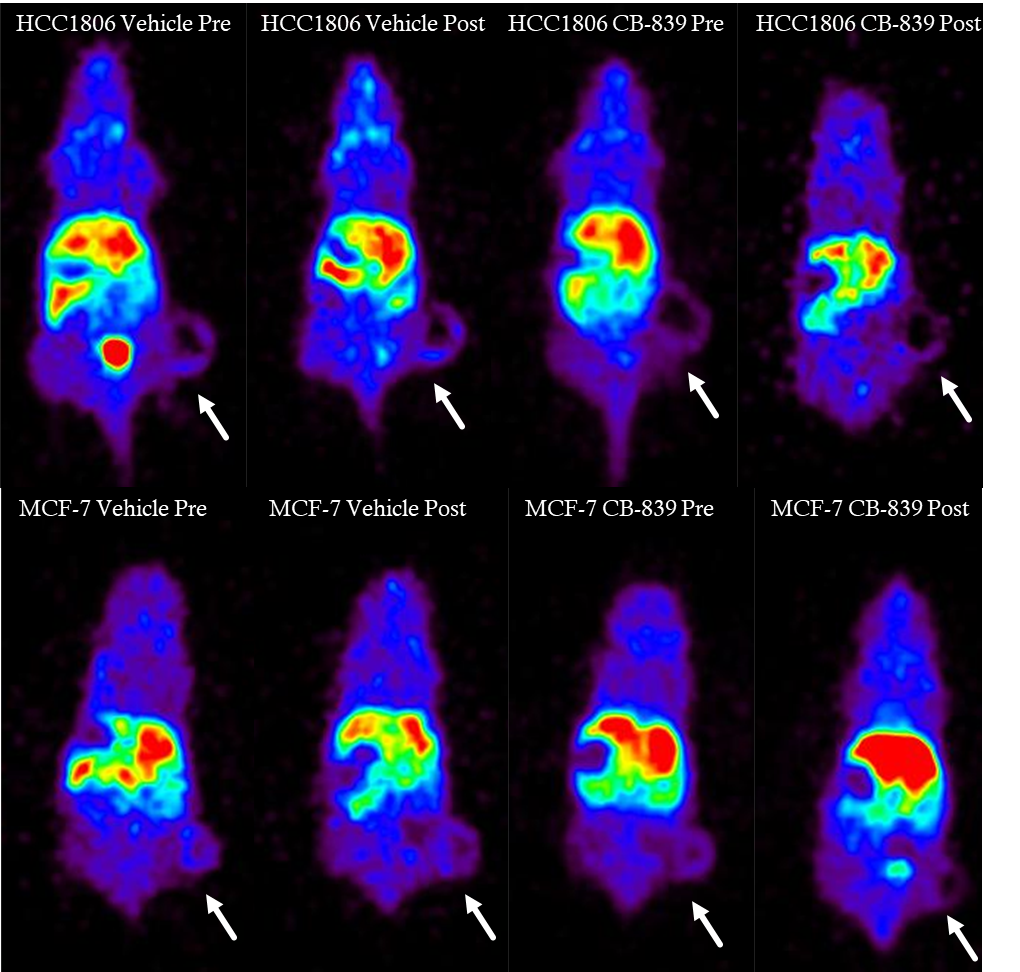


**Supplemental Figure 3:** Images of a representative mouse in each condition, taken at the last frame of the imaging experiment.


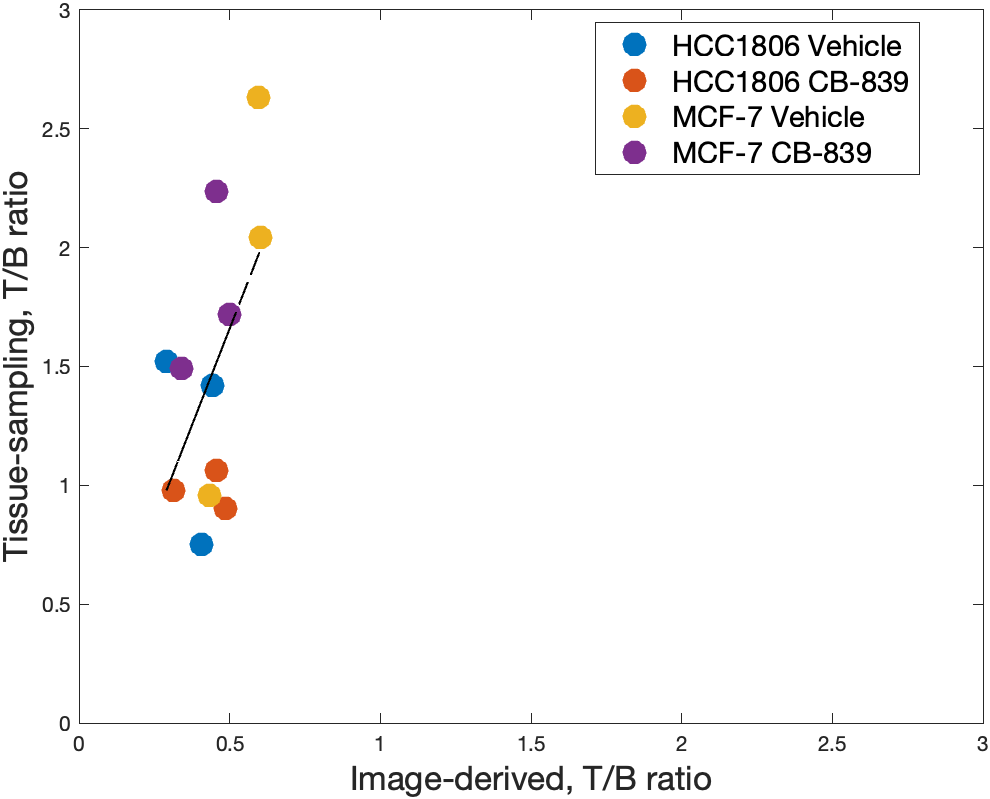


**Supplementary Figure 4:** Analysis of tumor-to-blood ratios comparing image-derived values to tissue-sampled values (R^2^=0.265). The three dots for each group represent that the sampling was performed at 10, 20 and 30 minutes.

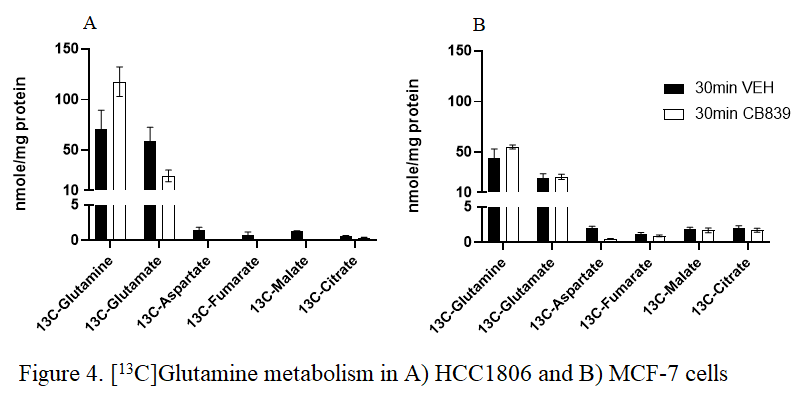


**Supplemental Figure 6:** [5-^13^C]GLN metabolism in A) HCC1806 and B) MCF-7 cells.

**Supplemental Figure 7:** Contribution of ^11^C-CO_2_ to total ^11^C signal in HCC1806 cells *in vitro* after 30 minutes of [5-^11^C]GLN administration (n=4)
